# Multi-institutional evaluation of a Pareto navigation guided automated radiotherapy planning solution for prostate cancer

**DOI:** 10.1186/s13014-024-02404-x

**Published:** 2024-04-08

**Authors:** Philip A Wheeler, Nicholas S West, Richard Powis, Rhydian Maggs, Michael Chu, Rachel A Pearson, Nick Willis, Bartlomiej Kurec, Katie L. Reed, David G. Lewis, John Staffurth, Emiliano Spezi, Anthony E. Millin

**Affiliations:** 1 Radiotherapy Physics Department, Velindre Cancer Centre, CF14 2TL Cardiff, Wales UK; 2 Northern Centre for Cancer Care, Cancer Services and Clinical Haematology, Newcastle upon Tyne, UK; 3 Worcester Oncology Centre, Worcestershire Acute Hospitals NHS Trust, Worcester, UK; 4 Translational and Clinical Research Institute, Faculty of Medical Sciences, Newcastle University Centre for Cancer, Newcastle University, Newcastle upon Tyne, UK; 5 School of Medicine, Cardiff University, Cardiff, Wales UK; 6 Velindre Cancer Centre, Medical Directorate, Cardiff, Wales UK; 7 School of Engineering, Cardiff University, Cardiff, Wales UK

**Keywords:** VMAT, IMRT, Automation, Treatment planning, Prostate cancer, Multi-institutional, Radiotherapy, Pareto, MCO, AI

## Abstract

**Background:**

Current automated planning solutions are calibrated using trial and error or machine learning on historical datasets. Neither method allows for the intuitive exploration of differing trade-off options during calibration, which may aid in ensuring automated solutions align with clinical preference. Pareto navigation provides this functionality and offers a potential calibration alternative. The purpose of this study was to validate an automated radiotherapy planning solution with a novel multi-dimensional Pareto navigation calibration interface across two external institutions for prostate cancer.

**Methods:**

The implemented ‘Pareto Guided Automated Planning’ (PGAP) methodology was developed in RayStation using scripting and consisted of a Pareto navigation calibration interface built upon a ‘Protocol Based Automatic Iterative Optimisation’ planning framework. 30 previous patients were randomly selected by each institution (I_A_ and I_B_), 10 for calibration and 20 for validation. Utilising the Pareto navigation interface automated protocols were calibrated to the institutions’ clinical preferences. A single automated plan (VMAT_Auto_) was generated for each validation patient with plan quality compared against the previously treated clinical plan (VMAT_Clinical_) both quantitatively, using a range of DVH metrics, and qualitatively through blind review at the external institution.

**Results:**

PGAP led to marked improvements across the majority of rectal dose metrics, with D_mean_ reduced by 3.7 Gy and 1.8 Gy for I_A_ and I_B_ respectively (*p* < 0.001). For bladder, results were mixed with low and intermediate dose metrics reduced for I_B_ but increased for I_A_. Differences, whilst statistically significant (*p* < 0.05) were small and not considered clinically relevant. The reduction in rectum dose was not at the expense of PTV coverage (D_98%_ was generally improved with VMAT_Auto_), but was somewhat detrimental to PTV conformality. The prioritisation of rectum over conformality was however aligned with preferences expressed during calibration and was a key driver in both institutions demonstrating a clear preference towards VMAT_Auto_, with 31/40 considered superior to VMAT_Clinical_ upon blind review.

**Conclusions:**

PGAP enabled intuitive adaptation of automated protocols to an institution’s planning aims and yielded plans more congruent with the institution’s clinical preference than the locally produced manual clinical plans.

**Supplementary Information:**

The online version contains supplementary material available at 10.1186/s13014-024-02404-x.

## Background

Automated radiotherapy treatment planning (AP) is an innovation that improves the quality and efficiency of plan generation when compared to traditional manual trial-and-error techniques [1]. Within the literature AP solutions can be separated into 3 broad categories:

1. Knowledge based planning (KBP): utilise algorithms trained on databases of historical treatment plans to predict parameters (e.g. dose volume histograms) that inform the optimisation of novel patients [2–6].
2. Constrained hierarchal optimisation (CHO): minimise clinical objectives in strict sequential order according to a predefined clinical ‘wish list’ [7, 8].
3. Protocol-based automatic iterative optimisation (PBAIO): automatically adapt parameters during the plan generation process, tailoring the optimisation to the individual patient [9–13].

Prior to automated plan generation all methods must be calibrated; a process that is critical in ensuring solutions are optimal and congruent with oncologists’ treatment wishes. At present two calibration methods are commonly employed. Simple trial-and-error, where AP parameters are iteratively adjusted manually based on the AP output, and machine learning where AP parameters/algorithms are trained on historical patient datasets. Trial-and-error is the predominant method used for PBAIO and CHO solutions, and machine learning for KBP solutions [1].

Whilst trial-and-error and machine learning yield clinically acceptable AP solutions, there are limitations of both approaches than can hinder the efficiency and optimality of the AP calibration. Machine learning generally requires large historical datasets (typically *n* = 100) [14], which may not be present for novel techniques or prescriptions, and calibrations are strongly dependent on the optimality and consistency of plans in the training dataset [15], which is not guaranteed. Additionally KBP trained with machine learning may still require considerable ‘tuning’ to deliver suitable solutions [16]. For trial-and-error, a key issue is that due to the high number of calibration variables and their possible permutations, efficient and intuitive exploration of different treatment options is not possible. Trial-and-error is analogous to traditional manual planning (albeit at the patient cohort level); an approach prone to inter-observer variability [17] and yielding plans that may not fully align with oncologists’ clinical aims [18]. The process is also inefficient with any change in calibration parameter requiring the generation of a new plan to assess the impact on the dose distribution.

We propose an alternative method for AP calibration, which utilises Pareto navigation techniques in place of trial-and-error or machine learning. The concept of Pareto navigation is as follows: (i) a plan is considered Pareto optimal when improvement of one objective/trade-off can only be made at the detriment of another (ii) for a given optimisation problem there is an infinite set of Pareto optimal plans, which define the ‘Pareto front’ (iii) in Pareto navigation the Pareto front is sampled (for all or a selected number of trade-offs) via generating a set of discrete Pareto optimal plans, the decision maker (e.g. oncologist or dosimetrist) then interactively explores the Pareto front using a navigation star [19] or sliders [20] to select the clinically optimum solution. When compared to traditional trial-and-error manual planning, on an individual patient basis Pareto navigation has been shown to improve planning efficiency by 70–90% [18, 21, 22] and yield solutions more congruent with the oncologists’ treatment aims [18]. It is therefore hypothesised that Pareto navigation presents an effective AP calibration alternative.

Recently the methodology of a fully automated PBAIO solution that was calibrated using Pareto navigation techniques (Pareto Guided Automated Planning (PGAP)) has been presented [23]. The solution was evaluated for prostate cancer patients with and without elective nodal irradiation at the local institution (Velindre Cancer Centre (VCC)), with results demonstrating superiority over manual planning [24]. However, in this initial implementation of PGAP, Pareto navigation was constrained to one trade-off (or dimension) at a time, which limited the effectiveness of the technique in exploring the Pareto surface.

The purpose of this work is to firstly present a new PGAP solution that implements a multi-dimensional Pareto navigation calibration interface and secondly to present results of a multi-centre validation of this solution in two external institutions.

## Methods

### Patient selection and planning protocol

For each institution (I_A_ and I_B_) 30 patients (60 in total) treated with prostate only radiotherapy during the period of 1st April– 30th June 2017 were randomly selected, with 10 and 20 patients allocated to a calibration and validation dataset respectively. Patients with hip prosthesis were excluded. Across both institutions patients were treated following the hypo-fractionated CHHIP trial protocol [25]; a simultaneous integrated boost technique delivering 60 Gy in 20#. The clinical goals associated with this protocol are presented in Table 1.

**Table 1:** CHHIP trial based clinical planning goals for I_A_ and I_B_

ROI Name	Dose Parameter	Goal
**Target and Max Dose Goals**		
All PTVs	D99%	≥ 95% of PTV prescription
PTV57.5 - PTV60	D50%	≥ 57.5 Gy
PTV48 - PTV57.5	D50%	≥ 48 Gy
PTV60	D1%	≤ 63.0 Gy
Patient Outline	D1.8 cm^3^	≤ 63.0 Gy
**OAR Goals**		
Rectum	V24.3 Gy	≤ 80%
Rectum	V32.4 Gy	≤ 70%
Rectum	V40.5 Gy	≤ 60%
Rectum	V48.6 Gy	≤ 50%
Rectum	V52.7 Gy	≤ 30%
Rectum	V56.8 Gy	≤ 15%
Rectum	V60.0 Gy	≤ 3%
Bladder	V40.5 Gy	≤ 50%
Bladder	V48.6 Gy	≤ 25%
Bladder	V60.0 Gy	≤ 5%
Femoral Heads	V40.5 Gy	≤ 50%
Bowel	V40.5 Gy	≤ 17 cm^3^

Patients were planned on a CT scan of 2 mm slice thickness, with prostate and up to 2 cm of proximal seminal vesicles (sv) delineated as targets; and rectum, bladder, femoral heads (I_B_ only) and bowel (I_B_ only) delineated as organs at risk (OARs). As per the CHHIP protocol the following planning target volumes (PTV) were generated, with the PTV’s nominal prescription in Gy defined by the nomenclature’s suffix: prostate expanded by 5 mm (0 mm posteriorly) and 10 mm (5 mm posteriorly) to form PTV60 and PTV57.5 respectively; and prostate + sv expanded by 10 mm to form PTV48.

The clinically delivered treatment plans (VMAT_Clinical_) were generated by the institutions using RayStation v5 (RaySearch Laboratories, Stockholm). Treatments were delivered on a Varian TrueBeam STx (Varian Medical Systems, Palo Alto) and an Elekta Agility (Elekta Ltd, Crawley) linac for I_A_ and I_B_ respectively. Automated plans (VMAT_Auto_) were generated at VCC using RayStation v4.99, a research release equivalent to v5. VMAT_Auto_ plans were generated using identical RayStation treatment planning machine models and arc configurations to VMAT_Clinical_ (single 6MV 360° VMAT arc). For I_B_, VMAT_Auto_ and VMAT_Clinical_ were normalised such that PTV60’s median dose equalled 60.0 Gy.

### Pareto guided automated planning

In this study PGAP was performed using EdgeVcc: a PBAIO automated planning solution developed at VCC and implemented in RayStation using python scripting. Full details of this PGAP solution are presented by Wheeler et al. [23], with the following providing a summary of the key aspects.

Prior to automated planning a site specific ‘AutoPlan protocol’ is created and a set of planning goals defined (Table 2). Planning goals are split into 3 priority levels: critical normal tissue goals (P_1_), target goals (P_2_) and normal tissue goals (P_3_). P_1_ and P_2_ generally represent a clinical protocol’s mandatory dose constraints and P_3_ all other trade-offs which are to be minimised. This approach is analogous to using constraints and trade-offs in standard Pareto navigation applications. No weighting factors (WF) are specified by the user, instead they are generated through two processes. For P_1_ and P_2_, WF are defined by hard coded constants (1000 and 250 for P_1_ and P_2_ respectively). For P_3_, balancing competing trade-offs is complex and difficult to define *a priori*. In this case WF are derived through the Pareto navigation calibration process.

**Table 2:** Final planning goals and weighting factors for both institutions

**Priority 1: Primary Conformality Goals (WF = 1000)**						
**ROI Name**	**Dose Parameter**	**Target (Gy)**	**Distance (cm)**			
PTV48	Dmax	46.8	1.5			
**Priority 2: Target Goals** **(WF = 250)**						
**ROI Name**	**Dose Parameter**	**Target** **(%**_**Presc,PTV**_ **)**				
PTV60	Dmin	98.7				
PTV60	Dmax	101.7				
PTV60	D50% max	99.5				
PTV57.5	Dmin	98.7				
PTV57.5	Dmax	102.5				
PTV48	Dmin	97.3				
PTV48	Dmax	104.9				
**Priority 3: Trade-off Goals (Standard)**						
**ROI Name**	**Dose Parameter**	**Target** **(Gy or %**_**Vol**_ **)**	**WF (I**_**A**_ **)**	**WF (I**_**B**_ **)**		
Rectum	V23.4 Gy (%)	0.0	3.5	3.5		
Rectum	V31.5 Gy (%)	0.0	3.5	3.5		
Rectum	V39.6 Gy (%)	0.0	**0.044**	**-**		
Rectum	V47.7 Gy (%)	0.0	**0.088**	**-**		
Rectum	V51.8 Gy (%)	0.0	29.9	29.9		
Rectum	V55.9 Gy (%)	0.0	**3.5**	**-**		
Rectum	Dmax (Gy)	60.0	0.586	0.586		
Rectum	Dmean (Gy)	5.0	5.84	5.84		
Bladder	V30.0 Gy (%)	0.0	0.316	0.316		
Bladder	V39.6 Gy (%)	0.0	0.316	0.316		
Bladder	V47.7 Gy (%)	0.0	0.316	0.316		
Bladder	V51.8 Gy (%)	0.0	0.316	0.316		
Bladder	V55.9 Gy (%)	0.0	**0.316**	**-**		
Bladder	Dmax (Gy)	54.0	0.316	0.316		
Bladder	Dmean (Gy)	5.0	3.73	3.73		
Bowel	V36.0 Gy (%)	0.0	**-**	**0.413**		
Bowel	V45.6 Gy (%)	0.0	**-**	**0.413**		
**Priority 3: Trade-off Goals (Dose Fall Off)**						
**ROI Name**	**Fall Off Type**	**High Dose Level (Gy)**	**Low Dose Level (Gy)**	**Dose Gradient** **(%**_**Presc**_ **cm** ^**− 1**^ **)**	**WF (I**_**A**_ **)**	**WF (I**_**B**_ **)**
External	Normal Tissue Falloff	60.0	30.0	50%	204	204
PTV57.5	Intra PTV Falloff	54.0	54.0	75%	**10.7**	**29.8**
PTV48	Intra PTV Falloff	54.6 (54.0)*	45.6	75%	29.8	29.8

*Abbreviations*: %_Presc, PTV_ = % of individual PTV prescription dose; %_Presc_ = % of overall treatment prescription; %_Vol_ = % volume of ROI, WF = weighting factor

*Notes*: Differences between I_A_ and I_B_ AutoPlan protocols are highlighted in bold. WF = ‘-’ indicates the planning goal was removed for the institution specific protocol. Priority 3 targets = 0.0 by default, but can be specified if desired. The target is dynamically adjusted during optimisation and therefore initial values have negligible impact plan quality, but may decrease planning time if correctly defined

*Value outside and inside parenthesis correspond to I_A_ and I_B_ respectively

Calibration is initially performed on a single patient. Firstly, a set of automated plans with differing P_3_ WF are generated using the PBAIO automated planning algorithms. These plans represent different AutoPlan calibration options, each with a different balancing of competing trade-offs that constitute a point on the Pareto front. The operator then navigates through these differently weighted P_3_ treatment options via a sliding interface. The clinically optimum position on the Pareto front, determined qualitatively by the operator, is selected and the WF associated with this navigated position stored in the AutoPlan Protocol. The result is a calibrated AutoPlan protocol, which is ready for testing or further refinement.

The PGAP solution is built on a PBAIO automated planning framework, where during optimisation the position and weight of P_3_ related optimisation objectives are iteratively updated. The position is adjusted to maintain a constant difference (δ) between the optimisation objective and its corresponding DVH parameter. For example, if a dose volume objective (DVO) of V23.4 Gy at 10.0% volume is defined and the resultant optimised dose yields a V23.4 Gy equalling 9.0%, the DVO volume target will be set to [9.0% - δ]. In terms of objective weight, this is dynamically updated such that the objective function’s value trends towards a target objective value. Utilising these two mechanisms within a PBAIO framework aims to both minimise OAR doses (via dynamic positioning) and ensure consistent trade-off balancing across all patients treated to the same clinical protocol (via dynamic weighting). This provides the potential for a Pareto navigation calibration on a single patient to yield a suitably calibrated AP solution for novel patients. In practice, especially for more complex sites with variable anatomy, it may be necessary to perform additional Pareto navigation on outlier patients (with weights typically averaged) to improve the solution’s robustness across the whole cohort.

**Fig. 1:**
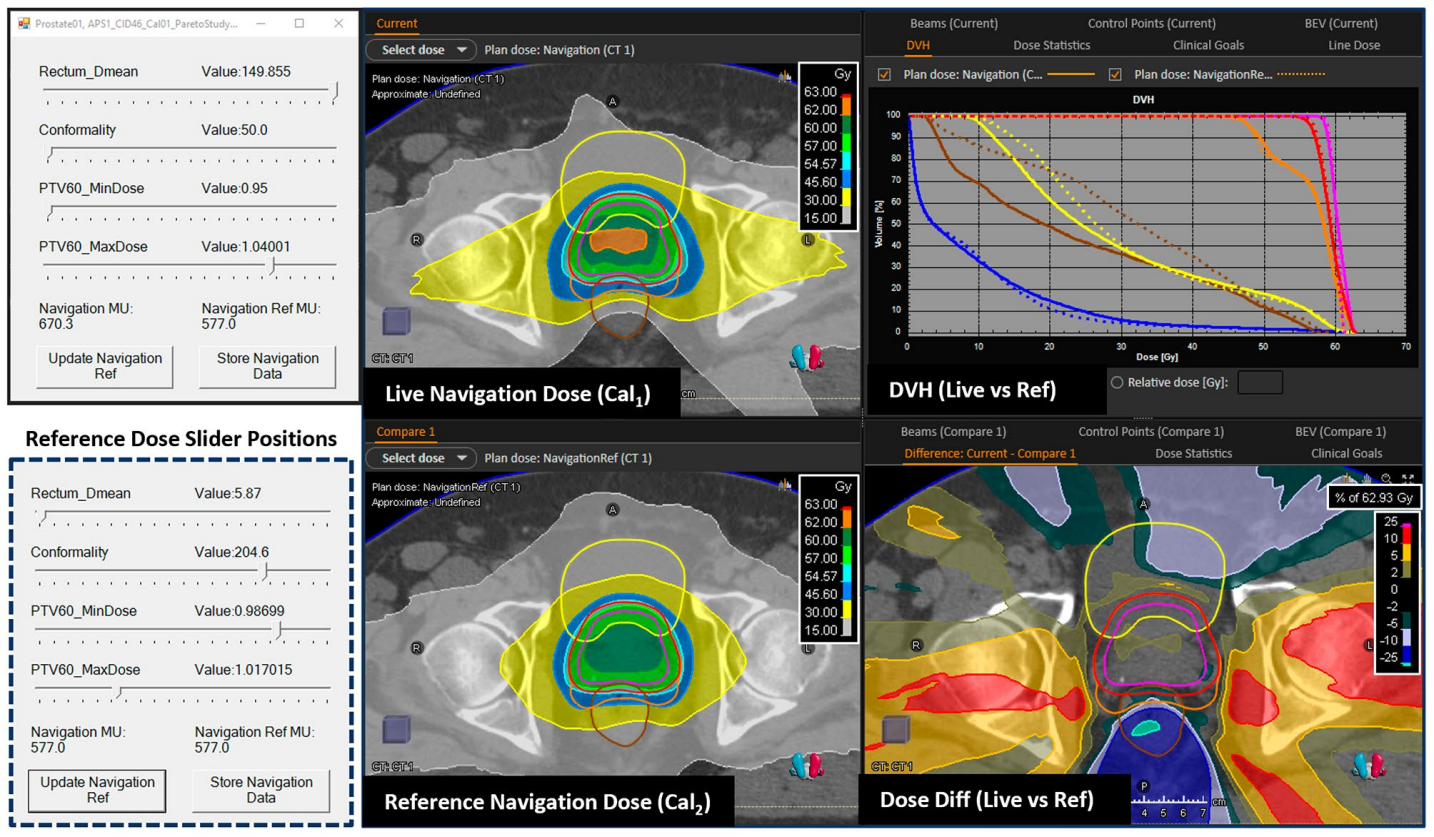
Pareto navigation calibration interface. Navigation is performed using the slider bars (top left), with the dose distribution (top centre) and DVH (top right– solid line) updated in real time within RayStation’s evaluation module. During navigation the operator can set the navigated distribution as a reference distribution (bottom centre) and DVH (top right– dotted line) to aid in the decision making. In this example the navigated position represents a solution where the rectum is spared at the expense of homogeneity and conformality (Cal_1_) with the reference distribution representative of the final calibration for I_A_ (Cal_2_). The corresponding Cal_2_ slider positions are provided for reference (bottom left) and isodose legends have been enhanced for clarity. ROIs: rectum (brown), bladder (yellow), external (blue), PTV60 (pink), PTV57.5 (red) and PTV48 (orange)

In previous work, calibration via Pareto navigation was performed through sequential navigation of one trade-off (or Pareto dimension) at a time. In this regard a Pareto dataset (typically containing 5 plans) was generated with varying WF applied to the given trade-off and all other WF held constant (or set to zero if unnavigated). The process was repeated until all trade-offs were navigated. In this work we present a fully customisable interface (Fig. 1), where any number of dimensions can be navigated in parallel, thereby providing the opportunity for full Pareto navigation. Furthermore, dimensions are not limited to a planning goal’s WF, but rather any of its parameters, enabling navigation, for example, of individual P_2_ target values such as PTV min dose.

For a given navigation the operator defines (via a config file) the dimensions to be explored and for each dimension the trade-off parameter values to be sampled during creation of the Pareto surface. Typically 3–5 parameter values are specified for each dimension. To populate the Pareto navigation dataset, a fully segmented treatment plan is generated (using the PBAIO framework) for all possible parameter value permutations across the different dimensions. The dataset is navigated in ‘parameter space’ using a slider interface with the navigated dose distribution estimated though linear interpolation of the neighbouring discrete Pareto plans using the navigated parameter values as the interpolation coefficients (see Wheeler et al. [23]). Whilst the interface allows for any number of dimensions to be navigated in parallel, there are computational limitations as the number of plans in the navigation dataset increases to the power of the number of dimensions. Pareto navigation is therefore typically limited to < 5 dimensions, with additional navigations performed sequentially until all trade-offs have been navigated.

### AutoPlan protocol calibration

Separate calibrations for both I_A_ and I_B_ were performed by VCC using the institution’s calibration patient cohort. Planning goals (Table 2) were based on CHHIP clinical goals (Table 1) and during calibration the balancing of trade-offs was informed by the corresponding VMAT_Clinical_ plan and collaborative discussions with the external institution.

### Demonstrating the utility of PGAP

To demonstrate the potential utility of PGAP, using the calibrated I_A_ protocol as a base, a multidimensional navigation consisting of the following four dimensions was generated for the first I_A_ calibration patient: PTV60 D_min_ (target parameter), PTV60 D_max_ (target parameter), rectum D_mean_ (WF parameter) and external normal tissue fall off (WF parameter). Using the navigation interface two different calibrations were selected (Fig. 1): Cal_1_, where the rectum was spared at the expense of homogeneity and conformality, and Cal_2_, where parameter values were set to nominally equal the final calibrated I_A_ protocol. For both Cal_1_ and Cal_2_ an automated plan was generated for all I_A_ calibration patients. Pareto front representations of PTV60 homogeneity index (HI_PTV60_), PTV48 Paddick’s conformity index (CI_PTV48_) [26] and rectum D_Mean_ were generated to demonstrate the propagation of differing calibrations to novel patients. This evaluation was undertaken at VCC after the multi-institutional study proper using an upgraded version of RayStation (8b research).

### Evaluative study design

For the evaluative study, VMAT_Auto_ plans were generated for all validation patients using the institution’s calibrated AutoPlan Protocol. Plan quality was quantitatively compared to VMAT_Clinical_ using: CHHIP dose metrics; PTV D98%, D2%, HI and CI; and OAR mean doses. Higher prescription PTVs were subtracted from lower prescription PTVs when reporting D98%, D2% and HI. Differences were assessed for statistical significance using a two-sided Wilcoxon signed rank test. Statistical testing was not performed where, following omission of tied values (i.e. where metrics equalled zero for both VMAT_Auto_ and VMAT_Clinical_), sample size was < 10. In addition, a blind qualitative comparison of VMAT_Auto_ and VMAT_Clinical_ was performed on-site at each external institution by a team consisting of a single oncologist and dosimetrist. During review the team would discuss the two plans under blind conditions and rank them in order of preference. Whilst the discussions were collaborative, it was permissible for the oncologist and dosimetrist to disagree on the final ranking.

## Results

### AutoPlan protocol calibration

Details of the calibrated AutoPlan Protocols are provided in Table 2. The final I_A_ protocol was used as a base for I_B_ following simplification (low weighted and similar planning goals removed). Due to substantial similarities in clinical preference between the two institutions only two key changes were made for the final I_B_ protocol: the addition of bowel goals and an increased intra-PTV dose fall-off WF.

### Demonstrating the utility of PGAP

**Fig. 2:**
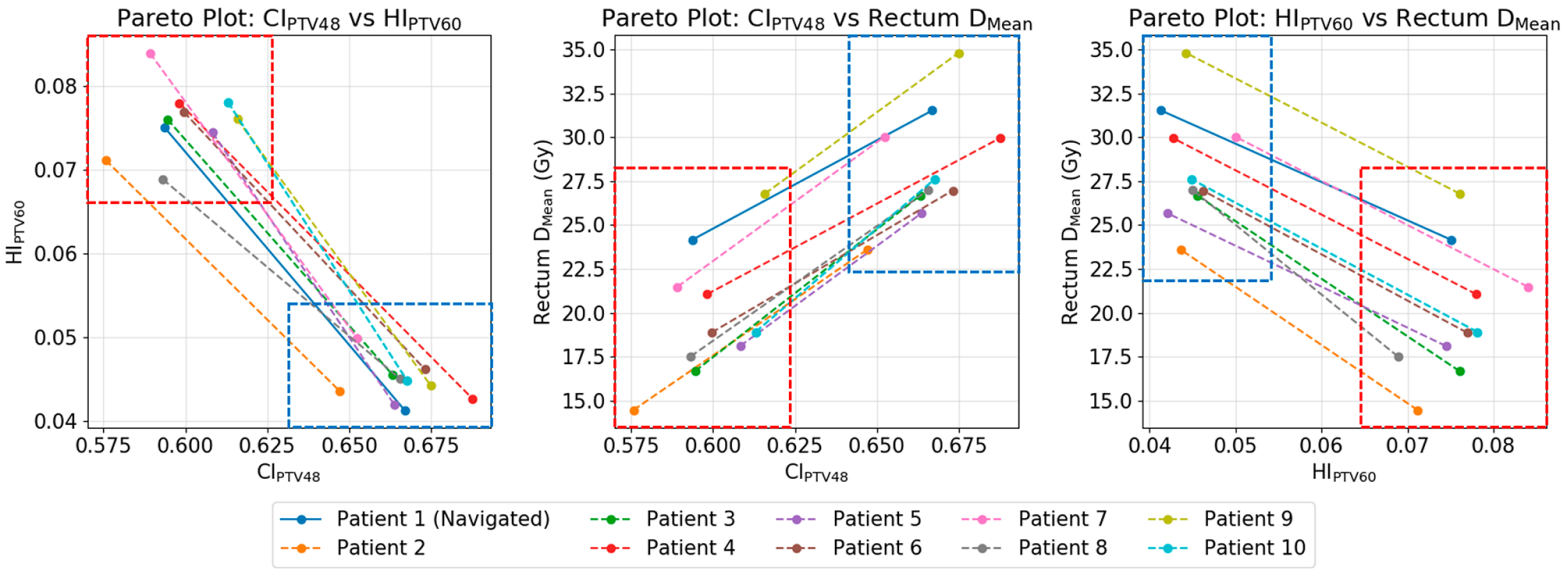
Pareto front representations of the three navigated trade-offs (rectum D_mean_, HI_PTV60_ and CI_PTV48_) demonstrating the dosimetric impact of two differently balanced calibrations (Cal_1_ & Cal_2_) on novel patients in the I_A_ calibration dataset. Data from the navigation patient (Patient 1) is presented for reference, with Cal_1_ and Cal_2_ data points encompassed by the red and blue boxes respectively

The Pareto front representations in Fig. 2 demonstrate how the two different calibrations propagated to novel patients. Across patients 2–10 there was a clear and consistent change in the balancing of automated plans between Cal_1_ and Cal_2_ with changes in rectum D_mean_, CI_PTV48_ and HI_PTV60_ of 8.7 Gy, 0.068, and − 0.031 respectively. This compares with changes of 7.4 Gy, 0.073 and − 0.034 respectively for the calibration patient (patient 1).

### Evaluative study

**Fig. 3:**
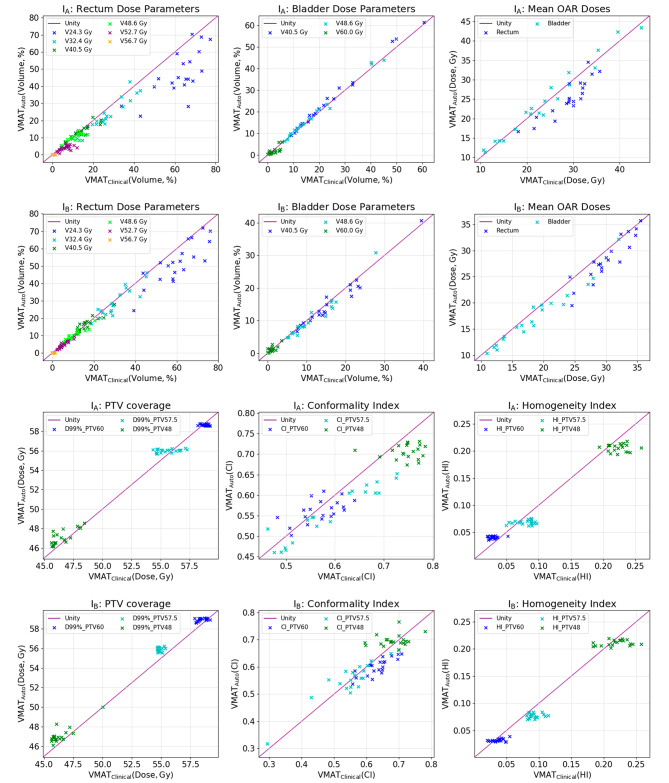
1–1 plots comparing VMAT_Auto_ and VMAT_Clinical_ across a range of OAR and PTV dose metrics for both institutions. Unity line is presented for reference and represents equivalence between the two techniques

Results of the evaluative study on the validation patient cohort are presented in Table 3, with Fig. 3 providing 1–1 plots comparing VMAT_Auto_ with VMAT_Clinical_ across a range of key OAR and PTV dose metrics. Across both institutions VMAT_Auto_ led to a statistically significant (*p* < 0.05) improvement across all but two rectal dose metrics (V_48.6 Gy_, V_52.7 Gy_). For I_A_, several reductions were substantial, with D_mean_ and V_24.3 Gy_ reduced by 3.7 Gy and 15.1% respectively. For I_B_ improvements were more modest [ΔD_mean_ = -1.8 Gy, ΔV_24Gy_ = -8.4%]. For bladder, VMAT_Auto_ led to a small but statistically significant detriment in low and intermediate dose level metrics for I_A_ [ΔV_40.5 Gy_ = + 1.3%, ΔV_48.6 Gy_ = + 0.6%] with the situation reversed for I_B_ [ΔV_40.5 Gy_ = -1.0%, ΔV_48.6 Gy_ = -0.7%]. VMAT_Auto_ led to a moderate reduction in bladder D_mean_ for I_B_ [ΔD_mean_ = -1.3 Gy].

**Table 3:** Dosimetric comparison of VMAT_Auto_ and VMAT_Clinical_ for institution A and B (mean ± standard deviation)

		Institution A			Institution B		
	Metric	VMAT_Auto_	VMAT_Clinical_	*p* value	VMAT_Auto_	VMAT_Clinical_	*p* value
PTV60	D98% (Gy)	**58.8 ± 0.1**	**59.0 ± 0.2**	**0.00**	**59.0 ± 0.1**	**58.7 ± 0.3**	**0.00**
	D99% (Gy)	**58.7 ± 0.1**	**58.8 ± 0.3**	**0.03**	**58.9 ± 0.1**	**58.5 ± 0.4**	**0.00**
	D2% (Gy)	**61.3 ± 0.1**	**60.8 ± 0.3**	**0.00**	61.0 ± 0.1	60.9 ± 0.4	0.68
	CI	0.562 ± 0.027	0.570 ± 0.041	0.31	**0.601 ± 0.031**	**0.636 ± 0.042**	**0.00**
	HI	**0.041 ± 0.002**	**0.030 ± 0.007**	**0.00**	**0.032 ± 0.002**	**0.037 ± 0.009**	**0.03**
PTV57.5	D98% (Gy)*	55.9 ± 0.1	55.5 ± 0.9	0.06	**55.7 ± 0.2**	**54.7 ± 0.2**	**0.00**
	D99% (Gy)	56.0 ± 0.2	55.6 ± 0.9	0.07	**55.9 ± 0.2**	**54.9 ± 0.2**	**0.00**
	D2% (Gy)*	**59.9 ± 0.1**	**60.2 ± 0.4**	**0.02**	60.3 ± 0.1	60.1 ± 0.5	0.18
	CI	**0.827 ± 0.013**	**0.862 ± 0.043**	**0.00**	**0.845 ± 0.013**	**0.863 ± 0.040**	**0.02**
	HI*	**0.068 ± 0.004**	**0.080 ± 0.014**	**0.00**	**0.077 ± 0.004**	**0.093 ± 0.009**	**0.00**
PTV48	D98% (Gy)*	**46.5 ± 0.3**	**45.8 ± 0.5**	**0.00**	**46.5 ± 0.2**	**45.5 ± 0.7**	**0.00**
	D99% (Gy)	**47.1 ± 0.7**	**46.5 ± 0.9**	**0.00**	**47.1 ± 0.8**	**46.4 ± 1.0**	**0.00**
	D2% (Gy)*	57.0 ± 0.4	57.3 ± 0.9	0.37	**57.2 ± 0.3**	**56.5 ± 0.9**	**0.01**
	CI	**0.707 ± 0.017**	**0.745 ± 0.033**	**0.00**	0.698 ± 0.021	0.683 ± 0.044	0.23
	HI*	**0.206 ± 0.007**	**0.224 ± 0.015**	**0.00**	0.211 ± 0.005	0.219 ± 0.020	0.17
Rectum	V24.3Gy (%)	**47.2 ± 13.0**	**62.3 ± 10.4**	**0.00**	**51.9 ± 11.6**	**60.3 ± 10.3**	**0.00**
	V32.4Gy (%)	**32.9 ± 12.1**	**44.1 ± 11.5**	**0.00**	**38.6 ± 10.9**	**43.6 ± 9.9**	**0.00**
	V40.5Gy (%)	**23.0 ± 9.1**	**26.5 ± 8.9**	**0.00**	**28.2 ± 8.8**	**29.7 ± 8.4**	**0.05**
	V48.6Gy (%)	13.6 ± 4.3	14.5 ± 5.3	0.17	15.9 ± 4.4	15.8 ± 5.0	0.79
	V52.7Gy (%)	9.5 ± 2.8	10.4 ± 3.8	0.16	10.1 ± 3.0	10.4 ± 3.9	0.50
	V56.8Gy (%)	**3.9 ± 1.6**	**6.4 ± 2.6**	**0.00**	**4.8 ± 2.0**	**5.5 ± 2.3**	**0.00**
	V60.0Gy (%)	**0.0 ± 0.0**	**0.3 ± 0.4**	**0.00**	**0.0 ± 0.1**	**0.3 ± 0.4**	**0.00**
	DMean (Gy)	**25.3 ± 4.7**	**29.0 ± 4.3**	**0.00**	**28.3 ± 4.1**	**30.1 ± 3.3**	**0.00**
Bladder	V40.5Gy (%)	**25.3 ± 14.7**	**24.0 ± 14.1**	**0.00**	**15.5 ± 7.4**	**16.4 ± 7.4**	**0.01**
	V48.6Gy (%)	**18.5 ± 11.3**	**17.9 ± 11.1**	**0.02**	**10.7 ± 5.9**	**11.4 ± 5.6**	**0.02**
	V60.0Gy (%)	1.8 ± 1.6	2.3 ± 1.7	0.07	1.2 ± 0.9	1.1 ± 0.8	0.65
	DMean (Gy)	24.3 ± 9.3	23.8 ± 9.2	0.20	**17.4 ± 5.3**	**18.7 ± 5.7**	**0.00**
Bowel	V40.5Gy (cm^3^)				0.8 ± 1.8	0.8 ± 1.6	n < 10
Femoral Head (Lt)	V40.5Gy (%)				0.0 ± 0.0	0.0 ± 0.1	n < 10
Femoral Head (Rt)	V40.5Gy (%)				0.0 ± 0.0	0.0 ± 0.0	n < 10
External	D1.8cm^3^ (Gy)	**61.2 ± 0.1**	**60.8 ± 0.2**	**0.00**	61.0 ± 0.1	61.0 ± 0.4	0.88
External	V5.0Gy (%)	**34.8 ± 6.1**	**32.5 ± 6.0**	**0.00**	27.2 ± 2.7	27.4 ± 2.8	0.17
Beam MU	MU	**637 ± 36**	**570 ± 37**	**0.00**	**739 ± 53**	**640 ± 118**	**0.01**
Plan Ranking vs VMAT_Clinical_	Plans Superior (%)	90%			65%		
	Plans Equivalent (%)	0%			30% (35%)		
	Plans Inferior (%)	10%			5% (0%)		

Results in bold indicate statistically significant differences (*p* < = 0.05). Dosimetrist plan rankings are provided in parenthesis where preference differs from the oncologist

CI: Paddick’s Conformity Index for the specified PTV.

HI: homogeneity index for the specified PTV

*Higher prescription PTV(s) subtracted from PTV when reporting

VMAT_Auto_ yielded moderate improvements in D_98%_ for PTV57.5 [I_B_ only, ΔD_98%_ = +1.0 Gy] and PTV48 [I_A_ ΔD_98%_ = +0.7 Gy, I_B_ ΔD_98%_ = +1.0 Gy], which did not result in a detriment in rectal doses. Significant but small differences were also observed for PTV60 D_98%_ [I_A_ ΔD_98%_ = -0.2 Gy, I_B_ ΔD_98%_ = +0.3 Gy]. D_2%_ was significantly increased for PTV60 [I_A_ only, ΔD_2%_ = +0.4 Gy], PTV48 [I_B_ only, ΔD_2%_ = +0.6 Gy] and deceased for PTV57.5 [I_A_ only ΔD_2%_ = -0.3 Gy]. Worthy of note was the reduction in the variation of HI across all study patients when planning with VMAT_Auto_, which was for all PTVs across both institutions (Fig. 3). In terms of conformality, VMAT_Auto_ led to moderate reductions in the CI index for I_A_ [ΔCI_PTV57.5_ = -0.035, ΔCI_PTV48_ = -0.039] and I_B_ [ΔCI_PTV60_ = -0.035, ΔCI_PTV57.5_ = -0.019]. This degradation was attributed to a higher prioritisation being placed on rectum dose reduction during calibration when compared with VMAT_Clinical_.

Upon blind review all plans were considered clinically acceptable. For I_A_ there was a clear preference towards VMAT_Auto_ with 90% considered superior to VMAT_Clinical_. For I_B_ this percentage dropped to 65% but the overall preference towards VMAT_Auto_ was maintained. Agreement between the oncologist and dosimetrist was very good with only one plan without a consensus decision. MU for VMAT_Auto_ was 12% and 15% higher than VMAT_Clinical_ for I_A_ and I_B_ respectively. This increase was not of concern to either institution.

## Discussion

In this study a PBAIO automated solution with a novel multi-dimensional Pareto navigation calibration methodology has been evaluated for prostate cancer in a multi-centre context. Results from the study demonstrated a clear clinical preference towards VMAT_Auto_ and provides supportive evidence on both the calibration method and underlying PBAIO framework that together form the PGAP solution.

This work builds upon the previous single institution study (performed at VCC [24]) in three key ways. Firstly, the updated calibration interface enabled multi-dimensional Pareto navigation, whereas the initial study was limited to a single dimensional proof of principle approach. This new method was fully congruent with the principles of Pareto navigation; enabling intuitive exploration of multiple competing trade-offs simultaneously. Secondly, the previous study provided no demonstration of the utility of PGAP; only presenting comparison of a single calibrated automated solution against manual planning. In this work a clear presentation of how different calibration choices propagate to novel patients via the PBAIO framework is provided (Fig. 2). Finally, a key challenge of any automated solution is demonstrating adaptability to the clinical requirements, techniques, and delivery machines of differing institutions. This study provides clear evidence that PGAP is a versatile solution, which can be successfully translated to independent external centres. Furthermore, with the vast majority of published studies being single institutional [1], this work helps to strengthen the evidence base on multi-institutional validations of automated solutions.

Within the literature there are limited examples on the utilisation of Pareto navigation to calibrate AP solutions and to our knowledge this work presents the first example where Pareto navigation is incorporated natively into the calibration process. The most relevant example is for KBP, where Pareto navigation was utilised by Miguel-Chumacero et al. [27] and Wall et al. [28] to improve the quality of the training dataset for head and neck, and prostate cancer respectively. This led to substantial reductions in OAR doses compared to a KBP model trained on the original manual planning based dataset. It is unclear if this is due to a conscious change in trade-off prioritisation or improving the optimality of the original manual plans. This approach, whilst promising, requires all training patients to be replanned, which is time consuming and presents a key barrier for practical implementation in the clinic. This is especially true for state-of-the-art dose distribution prediction solutions where training datasets are of the order of 100 patients [5]. In contrast the PGAP approach we developed can be calibrated through Pareto navigation on more limited patient datasets and is therefore ideal for rapid implementation of novel protocols or changes to clinical priorities due to emerging evidence.

The process of effective calibration is non-trivial; it requires an assessment of not only the clinical acceptability of a given calibration, but also the rate of change of competing dose metrics as the balancing of parameters is adjusted. For example, a detriment in CI of 0.05 may be acceptable if rectum D_mean_ reduces by 0.5 Gy but unacceptable for a 0.05 Gy reduction. It is our view that Pareto navigation is currently the only method that provides the operator with live access to this key information when calibrating an automated solution (via both the DVH and whole 3D dose distribution) and offers a clear alternative to machine learning and trial-and-error. Figure 1 illustrates the benefits of this approach, demonstrating how different treatment options can be interactively explored to identify the solution which best aligns with clinical preferences of the institution.

Successful PGAP implementation requires trade-off balancing of novel patients to be consistent with that selected during calibration. In our implementation, this function was fulfilled through building the solution on a PBAIO framework. This study provides evidence supporting this approach, firstly by demonstrating how trade-off balancing during calibration propagates effectively to novel patients (Fig. 2) and secondly through results of the blind review, which showed that PGAP yielded plans of high congruence with the institutions’ clinical preferences. Importantly, it is our view that a broad spectrum of PBAIO and CHO solutions presented in the literature also fulfil this requirement and therefore could benefit from integration of Pareto navigation into their calibration process.

The implemented approach does have limitations. Firstly, sampling the Pareto front using a simple exhaustive approach (plans generated for all parameter permutations) was computationally expensive and limited the practical number of Pareto dimensions per navigation to ≈ 4. Whilst, in this study it was not considered a significant constraint as many trade-offs were observed to be uncorrelated (e.g. CI_PTV48_ and rectum D_max_), it reduced the efficiency and elegance of the calibration process. Utilisation of more sophisticated sampling strategies [29] to reduce the computational burden would help increase the number of dimensions possible per navigation. Secondly, as is the case with all CHO and PBAIO solutions presented in the literature, a single AutoPlan Protocol was used across all study patients. Whilst resultant plans were on average superior to VMAT_Clinical_, utilisation of a single AutoPlan protocol assumes the clinically optimum balancing of competing trade-offs is consistent across individual patients, which may not be the case. It is recommended that further work evaluating per patient Pareto navigation vs. AP should be performed to explore the validity of this assumption.

In terms of the multi-centre evaluation a key observation during calibration was that, whilst the Pareto navigation interface enabled navigation of a wide range of differing trade-off options (Fig. 1), a solution which aligned reasonably closely to local clinical practice in terms of HI_PTV60_, CI_PTV48_ and modulation was selected by each institution. This was at the expense of further potential reductions in rectum D_Mean_ and reflected the institutions’ measured and proportional caution in selecting a solution, which if implemented would substantially change not only the planning method (automated from manual) but also the plan distribution and modulation for the whole treatment site. This trade-off prioritisation differed to VCC (where rectum D_Mean_ is prioritised over HI_PTV60_ & CI_PTV48_) and highlighted the importance of AP solutions having the functionality to allow full customisation of protocols to suit local requirements such that potential implementation barriers can be reduced.

As with the previously reported single institutional study of PGAP, this multicentre evaluation demonstrates superiority of automated planning over manual planning, both in terms of reduced rectum doses and clinical preference. This superiority was attributed to the improved alignment of trade-off balancing with clinical preference (particularly for CI vs. rectum D_mean_), and the PBAIO framework dynamically adjusting objectives to drive plans towards Pareto optimality. For I_A_, reductions in rectum D_mean_ were more substantial than I_B_ (3.7 Gy vs. 1.8 Gy respectively) due to their increased prioritisation of CI_PTV48_ for VMAT_Clinical_. This prioritisation was not congruent with the institution’s clinical preferences and was reflected in 90% of VMAT_Auto_ plans being preferred to VMAT_Clinical_ (compared to 65% for I_B_). Results (Fig. 3; Table 3) also highlighted a wide variation in the differences between VMAT_Auto_ and VMAT_Clinical_ both at an inter-patient and inter-institutional level. This was attributed to the inconsistencies associated with manual planning that have been widely reported in the literature [17, 30]. In comparison to a similar study [31] that evaluated a CHO approach across 4 institutions for prostate cancer our results are aligned, with that work also demonstrating overall superiority of VMAT_Auto_, with a median reduction in rectum D_Mean_ of 3.4 Gy (range [-4,12] Gy) as compared to 2.8 Gy (range [-1,7] Gy) in this study. Whilst direct comparison of the two approaches (PGAP/PBAIO vs. CHO) is not appropriate due to confounding factors such as differing planning systems, clinical protocols and the underlying quality of the manual comparators, this alignment adds strength to the findings by both authors that: (1) wide variations in the differences between VMAT_Auto_ and VMAT_Clinical_ are suggestive of inconsistencies in manual planning; and (2) AP solutions that seek Pareto optimality can yield substantial improvements in plan quality.

Finally, an interesting and unexpected outcome from this study was that once presented with results from both institutions, I_A_ adapted their manual planning practice to align closer with clinical preferences (i.e. prioritise rectum at the expense of CI_PTV48_). This led to a sustained reduction in rectum doses for clinical patients and highlighted the potential in utilising AP for cross-institutional audits to improve practice.

## Conclusions

A novel PGAP solution has been successfully validated against clinical practice for two external institutions. The multi-dimensional Pareto navigation calibration methodology enabled intuitive adaptation of automated protocols to an institutions’ individual planning aims without the requirement of large training datasets. Automated plans were more congruent with the institutions’ clinical preferences than manual plans and considered to represent a higher quality, more consistent and more efficient plan generation method.

### Electronic supplementary material

Below is the link to the electronic supplementary material.

Supplementary Material 1

## Data Availability

Evaluative study data is provided as a supplementary file. In addition, the datasets used and/or analysed during the current study are available from the corresponding author on reasonable request.
